# Digital and Mobile Health Technology in Collaborative Behavioral Health Care: Scoping Review

**DOI:** 10.2196/30810

**Published:** 2022-02-16

**Authors:** Khatiya Moon, Michael Sobolev, John M Kane

**Affiliations:** 1 Zucker Hillside Hospital Northwell Health Glen Oaks, NY United States; 2 Cornell Tech Cornell University New York City, NY United States

**Keywords:** collaborative care, integrated care, augmented care, digital health, mobile health, behavioral health, review

## Abstract

**Background:**

The collaborative care model (CoCM) is a well-established system of behavioral health care in primary care settings. There is potential for digital and mobile technology to augment the CoCM to improve access, scalability, efficiency, and clinical outcomes.

**Objective:**

This study aims to conduct a scoping review to synthesize the evidence available on digital and mobile health technology in collaborative care settings.

**Methods:**

This review included cohort and experimental studies of digital and mobile technologies used to augment the CoCM. Studies examining primary care without collaborative care were excluded. A literature search was conducted using 4 electronic databases (MEDLINE, Embase, Web of Science, and Google Scholar). The search results were screened in 2 stages (title and abstract screening, followed by full-text review) by 2 reviewers.

**Results:**

A total of 3982 nonduplicate reports were identified, of which 20 (0.5%) were included in the analysis. Most studies used a combination of novel technologies. The range of digital and mobile health technologies used included mobile apps, websites, web-based platforms, telephone-based interactive voice recordings, and mobile sensor data. None of the identified studies used social media or wearable devices. Studies that measured patient and provider satisfaction reported positive results, although some types of interventions increased provider workload, and engagement was variable. In studies where clinical outcomes were measured (7/20, 35%), there were no differences between groups, or the differences were modest.

**Conclusions:**

The use of digital and mobile health technologies in CoCM is still limited. This study found that technology was most successful when it was integrated into the existing workflow without relying on patient or provider initiative. However, the effect of digital and mobile health on clinical outcomes in CoCM remains unclear and requires additional clinical trials.

## Introduction

### Background

There are more people who could benefit from behavioral health services than can be served by the currently existing resources for care [1,2]. Mood and anxiety disorders are highly prevalent in the general population [3,4]. These disorders are disabling to individuals and burdensome to communities, resulting in increased service use, loss of productivity, and poorer outcomes for pre-existing medical conditions [5-7]. Common behavioral health problems are frequently treated in primary care settings because of the relative scarcity of behavioral health specialists in many areas [8,9]. However, primary care providers (PCPs) often lack the training and resources to manage these problems effectively, resulting in overstrained primary care practices and the potential for suboptimal care [8,10]. Novel approaches are needed to improve the scale, delivery, and cost efficiency of behavioral health care.

The collaborative care model (CoCM) aims to meet this vast need [11]. The CoCM is a well-established mode of treatment for common behavioral health disorders in primary care settings. Briefly, in this model, a PCP systematically screens patients for common behavioral health disorders and refers those in need to a behavioral health care manager (BHCM). The BHCM, typically colocated with the PCP, sees patients for an initial assessment and provides time-limited psychotherapy and ongoing evaluation. The BHCM liaises with a consulting psychiatrist who provides treatment recommendations. The psychiatrist may supervise multiple BHCMs at multiple primary care sites, thus significantly extending their reach. The BHCM tracks patient outcomes with regularly administered symptom rating scales (eg, the Patient Health Questionnaire-9 or the Generalized Anxiety Disorder-7 [12,13]) in conjunction with clinical evaluation. The BHCM communicates recommendations to the PCP, who prescribes any necessary medications and remains the clinician of record. This model is effective, widely regarded as the best practice, and has been adopted by many health systems across the world since its introduction in the 1990s [11,14-18].

Pitfalls along every aspect of collaborative care (CC) may contribute to unsuccessful implementation. For example, as the CoCM is a specialized multicomponent service, robust adoption requires provider training and stakeholder buy-in [19]. However, local expertise may be scarce. The colocation of the PCP and BHCM may also be a logistical challenge in some settings. To maximize reach, screening for behavioral health disorders within the primary care population must be systematic [20]; subsequent referrals to the BHCM should be streamlined in the clinical workflow. Monitoring symptoms within a large caseload requires efficient tracking mechanisms. The scope of interventions traditionally available to patients includes brief psychotherapy and limited pharmacotherapy [11]. Even under trial conditions, these options may not meet the needs of some patients [17]. Moreover, there are potential financial challenges to implementation [21].

Digital and mobile health technologies have the potential to support multiple components of CoCM [22]. Technologies such as mobile apps, wearable and ambient sensors, social media, and web-based platforms and devices can improve implementation by supporting provider training, patient screening and referral, monitoring, and treatment. For example, improving live and asynchronous communication technology can link novices with experts who can support the development of a CC program. Technologies can continuously collect interpretable patient data and potentially enable quick, flexible, and targeted care [23]. These resources can act as extenders, decreasing provider workload by automating tasks previously required of clinicians in the CoCM and supporting clinician decision-making by providing actionable clinical information in real time [22,24]. Digital and mobile health technologies can also support patients by providing education and real-time feedback from clinicians, reinforcing concepts learned in therapy, and tracking progress [22]. Emerging research has examined the potential of digital solutions for common behavioral disorders in primary care settings [25-27].

Technology has been recognized as important for the optimal functioning of the CoCM from an early stage. For example, clinicians and researchers recognized the necessity of using caseload registries to manage patient information, track outcomes, and have easy access to information from assessments and follow-up appointments [28-32]. In addition, telephone- and 2-way video-based remote care emerged in the 2000s as a viable method of providing CC [33-36]. Thus, there is extensive precedent and continuous interest in the use of technology to augment CC, which is unsurprising, given the model’s spirit of innovation and dissemination.

This review aims to summarize the current state of research into the ability of digital and mobile health to augment the CoCM, highlight important challenges and limitations, and explore areas for further investigation.

### Objective

The primary aim of this review is to synthesize the evidence available on digital and mobile health technology in CC settings.

## Methods

### Scoping Review

The topic of CC augmented with technology includes a wide variety of potential interventions, both patient-facing and provider-facing, and a wide variety of psychiatric disorders, including depression, anxiety, posttraumatic stress disorder (PTSD), bipolar disorder, and others. Thus, we chose to conduct a scoping review on this broad and emerging topic using the methodology recommended by Arksey and O’Malley [37] and refined by Levac et al [38]. To this end, we conducted six stages of review development: defining the research questions to broadly capture the relevant literature in this emerging field; balancing breadth and feasibility in the search strategy; identifying and selecting studies; extracting and charting data; and synthesizing and reporting the findings, including a forward-looking discussion that could guide future research or quality improvement efforts.

### Research Question

We attempted to answer 3 research questions with this scoping review. First, what digital and mobile health technologies have been studied in CC, and at what levels have they been implemented (patient- vs provider-facing)? Second, what is known about the acceptability and feasibility of digital and mobile health use in the CC context? Third, what, if anything, is known about the impact of these technologies on clinical outcomes in this setting?

### Search Strategy

We searched 3 web-based databases (Web of Science, MEDLINE, and Embase) during February 2020 with search terms related to CC (sometimes termed integrated care) and various technology interventions, including mobile apps, sensors, social media, and wearable devices. Scans of dark literature from Google Scholar were also completed. Scoping reviews on similar topics were consulted in the search strategy development [39,40]. Additional searches with the same search criteria were conducted in December 2020 and September 2021 to fully capture the status of this rapidly growing field of research. A full list of the search criteria is presented in Multimedia Appendix 1.

### Inclusion and Exclusion Criteria

Articles were determined eligible for inclusion if they described original research on digital and mobile health to augment the CoCM for the treatment of common behavioral health conditions. Novel technologies such as mobile apps, web-based platforms, ambient or wearable sensors, and social media were included. Technologies that are already well-established in the CoCM and in medicine broadly, such as electronic medical records, caseload registries, or telemedicine, were excluded. Opinion pieces, reviews, books, book chapters, protocols, and commentaries were excluded. Post hoc analyses of the trials included in this review were only included if they helped answer the questions posed by this review; otherwise, they were excluded. Articles written in languages other than English were also excluded. Studies were excluded if they recruited patients from a primary care population or other general medical populations that did not participate in a CoCM, as described previously in this paper. Studies in which substance abuse was the primary diagnosis were excluded.

### Study Screening Process

Owing to the large volume of articles obtained in the initial screening, the articles were divided and screened by 2 independent reviewers (KM and MS). In the initial stage of screening, articles were excluded based on the title and abstract. In the second stage, the remaining papers were excluded based on their full text. At the stage of full-text screening, conflicts were resolved by consensus between the reviewers.

## Results

### Summary of Studies

**Figure 1:**
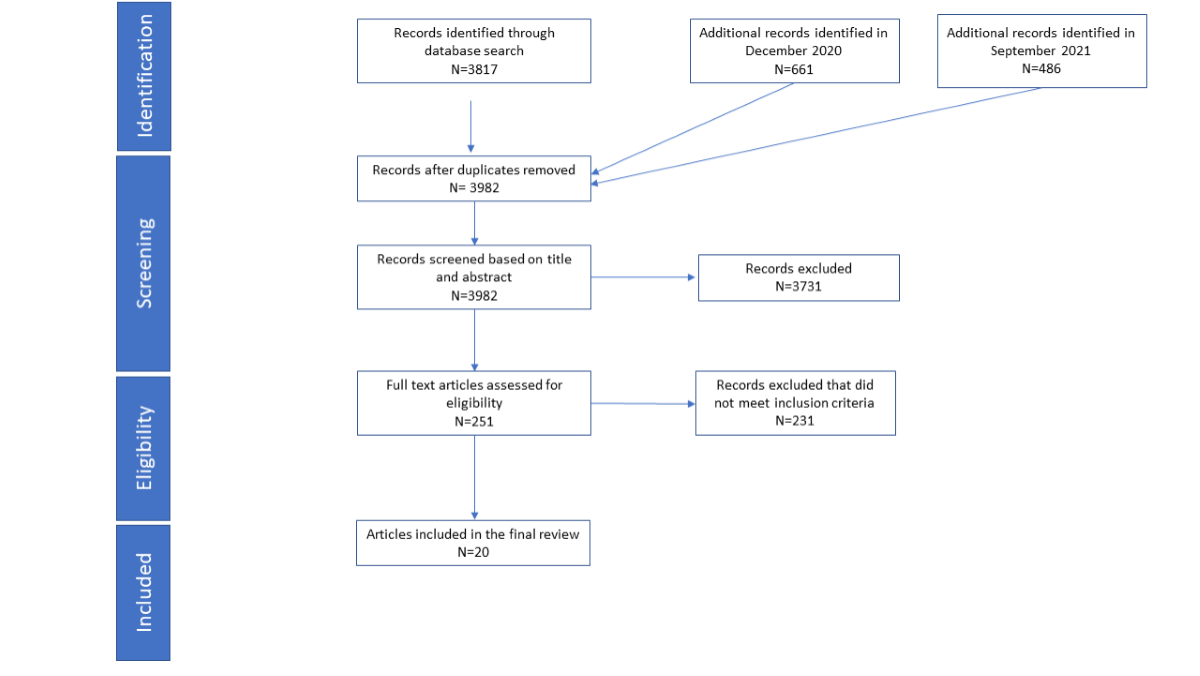
Study selection diagram.

The initial search identified 3817 reports; 661 additional reports were identified in December 2020, and 489 were identified in September 2021. The study selection process is summarized in Figure 1. A total of 20 studies were included in this review. The characteristics of the studies are summarized in Table 1, and more details on individual studies are provided in Multimedia Appendix 2 [15,35,41-58]. The 20 studies included a comparative effectiveness trial of an interactive voice recording (IVR) intervention in a safety net population and a series of post hoc analyses on the same study population [41-44], a randomized trial of an internet support group with an associated subgroup analysis [45,46], 30% (6/20) other randomized trials [35,47-52], a nonrandomized trial [53], 25% (5/20) implementation trials without a comparator [15,54-57], and a qualitative exploratory study [58].

**Table 1:** Characteristics of the included studies (N=20).

Characteristics					Studies, n (%)
**Study metadata**					
	**Study design**				
		Randomized trial	8 (40)		
		Quasi-experiment or nonrandomized trial with comparator group	2 (10)		
		Qualitative or mixed methods	6 (30)		
		Post hoc analysis	4 (20)		
	**Year of publication**				
		2018 or newer	15 (75)		
		2015-18	4 (20)		
		2014 or before	1 (5)		
**Population characteristics**					
	**Size**				
		≤10	1 (5)		
		10-50	5 (25)		
		50-200	2 (10)		
		200-500	4 (20)		
		>500	7 (35)		
		Not specified	1 (5)		
	**Diagnosis**				
		Anxiety disorders	4 (20)		
		Depressive disorders	14 (70)		
		Bipolar disorder	1 (5)		
		Posttraumatic stress disorder	3 (15)		
	**Patient- vs provider-facing**				
		Patient-facing	13 (65)		
		Provider-facing	3 (15)		
		Combination	5 (25)		
**Digital and mobile health intervention used to augment collaborative care**					
	SMS text messaging			3 (15)	
	Interactive voice recording			5 (25)	
	Mobile app			5 (25)	
	Web-based platform			7 (35)	
	Videoconferencing			1 (5)	
	Other			4 (20)	
**Outcome measured**					
	Adherence			3 (15)	
	Acceptability			7 (35)	
	Feasibility			9 (45)	
	Clinical improvement			10 (50)	
	Other			3 (15)	
**Comparator**					
	Collaborative care			4 (20)	
	Primary care			3 (15)	
	Collaborative care and primary care			4 (20)	
	Other			2 (10)	
	No comparator			7 (35)	

### Synthesis of Findings

#### Overview

We have summarized the results of our review in light of the research questions that we posed. We first describe which digital and mobile health technologies have been studied in CC and the nodes of the CC workflow that have been implemented. Next, we describe what is known about the acceptability and feasibility of digital and mobile health use in CC settings. Finally, we describe what is known about the impact of these technologies on the clinical outcomes in this setting.

#### Digital and Mobile Health Technologies in CC

Most studies identified by this review implemented or assessed multiple technologies at once rather than a single intervention. Of the 20 studies, 5 (25%) [15,50,53,54,57,58] assessed mobile apps, and 5 (25%) used a web-based platform [46,49-52]. Adewuya et al [47] and Jin and Wu [48] used SMS text messaging as a core feature of the intervention, although other investigators used SMS text messaging as an adjunctive tool [51,53,59]. Of the 20 research groups, 2 (10%) used IVR [49,60]. No studies identified in this review investigated social media, web-based search activities, or wearable devices.

The studies considered in this review encompassed all aspects of the CC workflow. Of the 20 studies, 3 (15%) used technology to augment BHCM training in the skills and implementation of the model [35,55,56]. One of the research groups [41-44,48] used technology to facilitate the screening of patients into CC by SMS text messages and IVR. Of the 20 studies, 1 (5%) described a decision support tool to triage patients into different levels of care [52]. Studies also used technology to facilitate measurement-based care by collecting clinical assessments [48,51,53,54]. Most studies provided participants with self-management modules and psychoeducational materials [15,35,46,49-51,53,54,57]. Several studies used technology to promote communication with the BHCM via asynchronous messaging [15,53,57]. Of the 20 studies, 1 (5%) used app-based therapy [57] and another assessed patient and provider attitudes toward app-based depression management [58]; 1 (5%) study used a web-based intervention in the form of web-based cognitive behavioral therapy and web-based group therapy [46]. Several studies had a patient-facing information collecting system that fed directly into a caseload management system [15,48,49,51,54,60].

#### Acceptability and Feasibility of Digital and Mobile Health in CC

The overall response to the technology presented in these studies was positive for both patients and providers. Patients enjoyed having the option of using technology for psychoeducation as therapy extenders and ways of communicating with their providers [15,41,49,50,54,57,58]. However, some of the technologies had limited sustainability, especially if they required sustained engagement from either patients or providers. Meglic et al [51] cited high dropout rates, and Bauer et al [54] had few participants who continued to use the app at 8 weeks. Less than half of the participants met the threshold for engagement in the study by Carleton et al [53]. Participation in IVR-based phone calls decreased after 6 months in the study by Vidyanti et al [44]. Fletcher et al [52] saw limited differences in intervention groups at 1 year. Participants cited privacy about the data collected in the apps as a concern in 15% (3/20) of studies [54,57,58]. Bhat et al [56] struggled with inconsistent attendance at meetings, which made it difficult for the provider training intervention to be effective. Meanwhile, staff cited concerns over increased workload if the technology intervention was not integrated into the usual medical record and required consulting a separate resource to see the results of data collection [54,58]. Providers also expressed concern over having little time to address the apps with patients and being uncomfortable or unfamiliar with the apps [57,58]. Usability and functionality of web- and app-based interventions were noted as concerns in the studies by Meglic et al [51] and Dinkel et al [58]. The potential cost of apps was noted as a barrier by Dinkel et al [58]. Meanwhile, studies that examined automated interventions such as IVR and automated SMS text messaging enjoyed higher levels of acceptability and feasibility [41,47,49]. The IVR-based screening trial for depression was noted to be cost-effective [42] and acceptable to patients [43]; providers reported feeling that they could spend more time focusing on clinical management if they knew that screening had already been completed [44]. Jin and Wu [48] found that participants with a high degree of depression stigma who were screened for depression using SMS text messaging were more likely to report certain symptoms when compared those screened with telephone interviews.

#### Clinical Outcome of Digital and Mobile Health in CC

Of the 20 studies, 8 (40%) studies examined clinical outcomes [35,41,45,46,49,51,53]. Of these 8 studies, 7 (88%) focused on depression or anxiety outcomes [41,45,46,49,51-53], whereas 2 (25%) focused on PTSD outcomes [35,50]. Of the 20 studies, the following 3 (15%) focused on patients with specific comorbid medical conditions: Zatzick et al [50] on combat-related injury, Wu et al [41] on diabetes mellitus, and Kroenke et al [49] on chronic musculoskeletal pain. Rollman et al [46] found that clinical outcomes were improved between the 2 intervention arms (which both used CoCM and technological interventions) compared with that of usual primary care. Zatzick et al [50] found a reduction in PTSD symptoms but not in depression symptoms in the information technology–enhanced CC group compared with that of usual primary care. However, these 2 studies had important limitations: they did not compare technology-enhanced CC with CC alone. Thus, it is not known whether the components of the CoCM or the technological intervention itself were associated with improvement. Of the 20 studies, 4 (20%) compared technology-enhanced CC with CC alone [41,49,51,53]. The study by Meglic et al [51] had small sample sizes but found an improvement in the clinical outcomes of the intervention group when compared with that of usual care. Both IVR-based studies (Wu et al [41] and Kroenke et al [49]) found modest improvements in clinical depression outcomes compared with CC alone. In the study by Carleton et al [53], which is the largest included study to investigate a mobile app, depression outcomes were similar between the app-augmented group and the usual CC group; however, the baseline depression severity was greater in the intervention group. Therefore, the authors speculated that the app may have improved outcomes beyond what might have been initially expected by facilitating communication and more frequent contact between patients and providers, given the baseline depression severity in this group. Fletcher et al [52] found that patients with both mild and severe illness showed improvement after using the decision support tool compared with usual care; however, these differences were modest.

## Discussion

### Principal Findings

In this scoping review, we investigated the use of digital and mobile health technology in CC settings. This study builds on previous research highlighting the potential of technology in improving behavioral health care [22,23,27].

Our results suggest that the implementation of digital and mobile health technology in CC is currently in its early stages in both clinical research and practice. For example, of the 20 studies, only 1 (5%) study using an app was a large trial [53]. Mobile apps were among the most novel technologies used by studies in this review; no studies using social media, wearable devices, ambient sensors, or other more innovative technologies were identified. Overall, there is more to learn about the use of technologies in this setting.

We believe that digital technology has the potential to support the delivery and scale of the CoCM by mitigating several common challenges to their effective implementation, ranging from provider training, patient screening and referral, monitoring and treatment, and sustainability of the practice. We also believe that the CoCM is especially suited to absorb such changes because of its forward-looking, team-based, and measurement-guided approach. We have used the results of our review to scope future directions for augmenting CC with digital and mobile health technologies focusing on provider training, screening, monitoring, treatment, and sustainability.

### Future of Digital and Mobile Health Technologies in Collaborative Care

#### Training and Adoption

Despite widespread recognition of the merits of the CoCM, adoption may continue to lag in part because of the lack of local expertise and provider training in the implementation of this complex, multicomponent service [11,19]. Of the 20 studies identified in this review, 3 (15%) addressed adoption and quality improvement challenges from several angles: remote coaching with videoconferencing, web-based self-guided modules for providers, and telephone-based training in behavioral health skill delivery [35,55,56]. These data suggest that a combination of premade self-guided materials and remote live coaching by experts can mitigate the need for local expertise in CC. The availability of these strategies, and, in the future, more robust evidence for their success, can also mitigate stakeholder hesitancy. In our review, longitudinal remote coaching was shown to experience lagging attendance at regularly scheduled coaching meetings that hindered clinical progress [56]. Digital tools such as asynchronous chat may alleviate this by encouraging providers to troubleshoot challenges in real time. In the future, other strategies, such as automated monitoring of caseload registries to measure faithfulness to the model, can further guide quality improvement and adoption efforts.

#### Screening and Referral

Increasing access to behavioral health care through systematic screening and referral is a core mission of the CoCM, one that technology has a great potential to support. Of the 20 studies, our review identified 1 (5%) research group that successfully used SMS text messaging and IVR to scale the screening of behavioral health disorders [44,48,61] and 1 (5%) study that used a patient-completed decision support tool to triage patients to different levels of care within an integrated, stepped-care system [52]. Surprisingly, most of the reviewed studies did not use technology for screening. However, the available data suggest that IVR, SMS text message, and other remote strategies can provide several benefits compared with traditional screening methods. For example, they could allow enrollment into the CoCM via the web or phone without attending a physical clinic, expanding access to people with mobility and engagement challenges [44,62]. They met the needs of a diverse population by being adapted relatively easily to languages other than English. Automated screening freed up provider time to address patient concerns [44]. Finally, as shown by Jin and Wu [48], automated screening may even be preferable because of its potential to identify individuals who may be unlikely to report symptoms in a traditional clinical interview. One of the advantages of IVR and SMS text messaging is that they do not require the patient to install any software or receive any education on the use of the technology, such as might be required by a mobile app or sensing device. Therefore, these tools may be especially well-suited to the screening process.

Although much further into the future, technology has the potential to facilitate screening by using digital biomarkers rather than self-report [63]. For example, the diagnosis of behavioral health disorders can be automatically assessed using speech processing and voice biomarkers. This technology can intuitively fit with existing voice technologies such as IVR [64]. For such data to be clinically meaningful, digital phenotypes of common behavioral health disorders will need to be well-established and validated; such research is currently in its infancy [65,66]. Although the CoCM was initially designed for depression, our review suggests that a diversity of disorders can be addressed in this context [17,50,55,67]. Automation can facilitate this by screening for a wider range of common behavioral disorders than is currently feasible in traditional, in-person, provider-administered screening processes [68]. This can then facilitate more targeted referral and earlier interventions [52]. Taken together, integrating these diverse data sources can enhance multimodal assessment and diagnosis of behavioral health disorders.

#### Remote Monitoring

Another core CoCM feature is its reliance on measurement-based care and systematic monitoring to guide clinical decisions. Our results suggest that technology can support this, particularly with the use of mobile apps, which can solicit symptom rating scales from patients at regularly set intervals [53]. This method supports measurement-based care by allowing a much more frequent collection of patient-reported outcomes than is currently possible in the interval clinical assessment model. If funneled into the electronic medical records, these data can then generate alerts to PCPs that would trigger an intervention from the BHCM if indicated [46]. Our results reinforce the idea that without integration into existing medical records and workflows, these data can actually be burdensome to staff, who would now have to consult multiple platforms to obtain clinical information [15,58]. Measurement-based care can be further enhanced by technology that allows for asynchronous communication between patients and the BHCM. Chat communication can reinforce therapeutic alliances, increase adherence to symptom reporting, and reduce the likelihood of treatment failure, thus improving clinical outcomes [22,57,58].

A future direction for digital and mobile health technologies in measurement-based care involves unobtrusive monitoring with connected devices, in particular mobile phones and wearable devices [69]. Patient-generated data can help measure clinical variables such as activity, sleep, and socialization, supporting measurement-based care and clinical decision-making and ultimately delivering personalized interventions when needed [66,70,71]. To date, these technologies have not been investigated as part of the CoCM but provide opportunities for future research and practice.

#### Treatment

Digital and mobile health technology can facilitate and scale treatment as part of the CoCM. For example, providing psychoeducational material is a basic intervention that was implemented in most of the studies in this review [15,35,46,49-51,53,54,57]. Mobile apps can allow the BHCM to monitor and nudge patients to engage with psychoeducational materials or provide automatic reminders. Higher engagement can be achieved by personalizing the technological solutions to deliver the right treatment, in the right amount, and at the right time [72-74]. This would require future research to identify the pathways for personalization. Most of the studies in this review showed improvement in clinical outcomes; however, most also implemented multiple technologies at once. To help personalize treatment, future research can more systematically identify therapeutic elements that directly relate to outcomes.

Digital and mobile health technologies can also help to facilitate more active treatment modules. For example, our review identified the preliminary use of cognitive behavioral therapy–based web-based treatments as part of technological implementation in CC [57,75]. Although a large number of therapy-based apps exist in commercial marketplaces, there is a dearth of research evaluating these apps in clinical practice [76,77]. If research were to show that these tools have meaningful clinical applications, their use could significantly scale the ability of CC to provide psychotherapy as treatment. There is also an opportunity to explore more novel treatment options that include virtual reality and other digital therapeutics and mindfulness-based interventions [27]. Future research should uncover which of these potential technologies are feasible and effective in CoCM settings.

### Sustainability and Challenges in Implementation of Digital and Mobile Health Technology

Research suggests that the implementation of digital and mobile health technologies in behavioral health care requires the addition of a new team member—a digital health coach—who might help patients and providers navigate digital interventions [78]. This can pose a challenge to the cost-effectiveness of digital technology. The structure of the CoCM has an advantage over standard primary care settings because of the existence of the BHCM, who could potentially act as a digital health navigator, and because of the infrastructure that already exists for collaboration between team members in this model.

There are several challenges in the implementation of digital and mobile interventions in CC. To provide evidence for efficacy, clinical outcomes in technology-augmented care should be superior to the clinical outcomes of traditional CoCM. The most comprehensive study of app-augmented care to date, by Carleton et al [53], found comparable effectiveness for both traditional (46%) and augmented CC (47%), as measured by improvement in Patient Health Questionnaire-9 scores [53]. To compensate for the lack of improvement in clinical outcomes, augmented care can be designed to save the cost of care and provider time. Currently, there is a relative lack of evidence on the effect of augmented care on cost and time, with only 5% (1/20) of the studies in this review addressing this issue [42]. In fact, digital and mobile health technologies may come with an additional cost to the providers as they require investment in the technology itself and in the implementation of its use. From this perspective, relatively low-cost interventions such as SMS text messaging and IVR, such as those implemented by Kroenke et al [62], Wu et al [61], and Adewuya et al [47], may be preferred.

Another common challenge in mobile health technology is the lack of engagement and high attrition rates, as demonstrated in several of the reviewed studies [15,53,56]. In augmented CC, the introduction of a mobile app or other technology for remote monitoring and treatment will require compliance with assessment modules and engagement with psychoeducational materials and chat communication. Our results show difficulties with engagement, especially in technologies that were not automated and required user-initiated actions to work [15,44,51]. In initial implementations, encouraging engagement and preventing attrition can become a function of the BHCM or digital health navigator. Future research should address these challenges through the lens of implementation science.

Finally, implementation of many of the discussed technologies requires addressing challenges with legal, ethical, and privacy concerns about the use of these data at both the patient and provider levels. This is a common challenge in technology, especially in the clinical context of behavioral health. Our review identified early reports of privacy concerns with regards to tracking [49,52]. These concerns might be amplified when additional tracking technologies for remote monitoring and treatment are introduced. Therefore, novel strategies must be implemented with the utmost concern for privacy in a cooperative and transparent manner.

### Limitations

Our review has several limitations. Methodologically, we did not use a librarian as part of the search strategy or calibration of the exclusion and inclusion criteria. Although improving the feasibility of our review, these strategies may have limited the breadth of our search and screening process. In a few years, as more technologies are implemented in CoCM, we expect a systematic review of the literature to be conducted to assess the evidence. In this review, we focused on the treatment of common behavioral health disorders in the CC setting specifically because of the unique approach and structure of this model. Therefore, we excluded studies of digital and mobile health technologies in related settings, such as primary care and substance abuse treatment. Nevertheless, as digital and mobile health technologies are used both in primary care and substance abuse, we drew and built on this research in our discussion.

### Conclusions

The use of digital and mobile health technologies in the CoCM is still limited. Digital technology was the most successful when it was integrated into the existing workflow without relying on the patient or provider initiative. The effect of digital and mobile health on clinical outcomes in CoCM remains unclear and requires additional clinical trials. To advance the use of digital and mobile health in CoCM, we have introduced a forward-looking discussion for augmenting CC with a focus on improving access to care, remote patient monitoring, and enhancing treatment.

## Appendix

Multimedia Appendix 1 Search terminology.

Multimedia Appendix 2 Summary of selected studies.

## Abbreviations

BHCM: behavioral health care manager
CC: collaborative care
CoCM: collaborative care model
IVR: interactive voice recording
PCP: primary care provider
PTSD: posttraumatic stress disorder
