# Supplementary material for: Digital and Mobile Health Technology in Collaborative Behavioral Health Care: Scoping Review
Source: JMIR Ment Health. 2022 Feb 16;9(2):e30810. doi: 10.2196/30810 (PMC8892315; doi:10.2196/30810)
Supplement: Multimedia Appendix 1 [file mental_v9i2e30810_app1.docx]

## Appendix 1: Search terminology

**Web of Science**

((ts=("collaborative care" OR "integrated care" OR ("primary care" AND psych*) OR ("primary care" AND "mental health") OR ("primary care" AND depress*) OR ("primary care" AND anxiety) OR ("primary care" AND "behavioral health") OR "integrated behavioral health" or "behavioral health integration")) AND (ts=("mobile health" OR mhealth OR technolog* OR "mobile app*" OR "social media" or facebook OR twitter OR sensor OR wearable* OR registr* OR mHealth* OR m-Health* OR smartphone* OR iPhone* OR android OR "cell phone*" OR cellphone* OR "cellular phone*" OR "mobile phone*" OR "mobile app*" OR "mobile device*" OR "mobile technolg*" OR tablet* OR "mobile tablet*" OR "electronic tablet*" OR iPad* OR iPod* OR "wearable technolog*" OR "consumer wearable*" OR (commercial* AND wearable*) OR "fitness tracker" OR "activity tracker" OR fitbit OR "smart watch*" OR smartwatch*))

**Medline**

("collaborative care" OR "integrated care" OR ("primary care" AND "mental health") OR ("primary care" AND anxiety) OR ("primary care" AND "behavioral health") OR "integrated behavioral health” OR "behavioral health integration") AND ("mobile health" OR "mobile app*" OR "social media" OR facebook OR twitter OR sensor OR wearable* OR registr* OR mHealth* OR m-Health* OR smartphone* OR iPhone* OR "cell phone*" OR cellphone* OR "cellular phone*" OR "mobile phone*" OR "mobile app*" OR "mobile device*" OR "mobile technolog*" OR tablet* OR "mobile tablet*" OR "electronic tablet*" OR iPad* OR iPod* OR "wearable technolog*" OR "consumer wearable*" OR (commercial* AND wearable*) OR "fitness tracker" OR "activity tracker" OR fitbit OR "smart watch*" OR smartwatch*)

**Google Scholar**

("collaborative care" OR "integrated care" OR ("primary care" AND "mental health") OR ("primary care" AND anxiety) OR ("primary care" AND "behavioral health") OR "integrated behavioral health” OR "behavioral health integration") AND ("mobile health" OR "mobile app*" OR "social media" OR facebook OR twitter OR sensor OR wearable* OR registr* OR mHealth* OR m-Health* OR smartphone* OR iPhone* OR "cell phone*" OR cellphone* OR "cellular phone*" OR "mobile phone*" OR "mobile app*" OR "mobile device*" OR "mobile technolog*" OR tablet* OR "mobile tablet*" OR "electronic tablet*" OR iPad* OR iPod* OR "wearable technolog*" OR "consumer wearable*" OR (commercial* AND wearable*) OR "fitness tracker" OR "activity tracker" OR fitbit OR "smart watch*" OR smartwatch*)

**EMBASE**

TI ("collaborative care" OR "integrated care" OR ("primary care" AND "mental health") OR ("primary care" AND anxiety) OR ("primary care" AND "behavioral health") OR "integrated behavioral health” OR "behavioral health integration") AND ("mobile health" OR "mobile app*" OR "social media" OR facebook OR twitter OR sensor OR wearable* OR registr* OR mHealth* OR m-Health* OR smartphone* OR iPhone* OR "cell phone*" OR cellphone* OR "cellular phone*" OR "mobile phone*" OR "mobile app*" OR "mobile device*" OR "mobile technolog*" OR tablet* OR "mobile tablet*" OR "electronic tablet*" OR iPad* OR iPod* OR "wearable technolog*" OR "consumer wearable*" OR (commercial* AND wearable*) OR "fitness tracker" OR "activity tracker" OR fitbit OR "smart watch*" OR smartwatch*)

OR

Abstract ("collaborative care" OR "integrated care" OR ("primary care" AND "mental health") OR ("primary care" AND anxiety) OR ("primary care" AND "behavioral health") OR "integrated behavioral health” OR "behavioral health integration") AND ("mobile health" OR "mobile app*" OR "social media" OR facebook OR twitter OR sensor OR wearable* OR registr* OR mHealth* OR m-Health* OR smartphone* OR iPhone* OR "cell phone*" OR cellphone* OR "cellular phone*" OR "mobile phone*" OR "mobile app*" OR "mobile device*" OR "mobile technolog*" OR tablet* OR "mobile tablet*" OR "electronic tablet*" OR iPad* OR iPod* OR "wearable technolog*" OR "consumer wearable*" OR (commercial* AND wearable*) OR "fitness tracker" OR "activity tracker" OR fitbit OR "smart watch*" OR smartwatch*)
