# Supplementary material for: Digital and Mobile Health Technology in Collaborative Behavioral Health Care: Scoping Review
Source: JMIR Ment Health. 2022 Feb 16;9(2):e30810. doi: 10.2196/30810 (PMC8892315; doi:10.2196/30810)
Supplement: Multimedia Appendix 2 [file mental_v9i2e30810_app2.docx]

## Appendix 2: Summary of selected studies

| **Reference** | **Population** | **Study Design** | **Primary outcomes** | **Digital and Mobile Health Intervention** | **Comparison** |
| --- | --- | --- | --- | --- | --- |
| Adewuya et al. , 2019 [47] | 895 adults with depression | Randomized trial | Adherence | Text message appointment reminders, text message psychoeducation, and automated voice message after missed appointments to augment collaborative care | Collaborative care alone |
| Bauer et al, 2018. [15] | 10 adults with bipolar disorder and/or PTSD | Implementation trial without a comparator | patient and provider satisfaction | mobile app linked to a registry | -- |
| Bauer, Iles-Shih et al., 2018 [54] | 17 adults with depression and/or anxiety | Implementation trial without a comparator | Acceptability and feasibility | Mobile app that collects sensor data, symptom rating scales, and provides psychoeducation modules | -- |
| Bauer, Jakupcak, et al., 2020 [55] | 34 BHCMs in 12 clinics | Implementation trial without a comparator | multiple | Behavioral activation training through online self-guided materials, videoconferencing workshops, telephone-based role play | -- |
| Bhat et al. 2020 [56] | Unknown number of team members in 3 clinics | Implementation trial without a comparator | acceptability and feasibility | Longitudinal remote coaching | -- |
| Carleton, et al., 2020 [53] | 807 adults with depression | Non-randomized trial | Clinical outcomes, engagement with the app, appointment follow up | Mobile app used to augment an existing collaborative care model within a large health system | Collaborative care alone |
| Dinkel, et al., 2021 [58] | 15 behavioral health providers and staff and 17 patients | Qualitative case study | feasibility | Mental health apps for depression | -- |
| Engel et al. , 2016 [35] | 666 veterans with PTSD | Randomized trial | PTSD and depression symptoms | Telemedicine-based collaborative care + telephone-based BHCM training, online CBT self-management course, and caseload registry | collaborative care alone |
| Fletcher et al., 2021 [52] | 834 adults with depression or anxiety | Pragmatic stratified randomized controlled trial | Psychological distress | Online decision support tool that predicts prognosis and personalizes recommendation for treatment | Usual care |
| Geramita et al., 2018 [45] | 302 adults with depression and/or anxiety | Post-hoc subgroup analysis of the ISG+CCBT cohort described in Rollman et al. | Quality of life, depression and anxiety symptoms, durability | Participants were stratified by how engaged they were with the ISG | -- |
| Hay et al., 2018 [42] | 1406 adults with type 2 diabetes in a safety-net clinic system with comorbid depression | Post-hoc cost-effectiveness analysis | Cost per QALY | IVR telephone based clinical screenings and follow up assessments | Collaborative care alone and usual primary care |
| Hoffman et al., 2019 [57] | BHCMs and 35 adults with depression and/or anxiety | Implementation trial without a comparator | Acceptability and feasibility | mobile health toolkit including multiple apps for therapy, meditation, psychoeducation | -- |
| Jin et al., 2020 [48] | 206 adults with diabetes mellitus and comorbid depression | Randomized trial | Validity | Text-message depression and anxiety screening in a primary care population with access to collaborative care | Interview-based screening |
| Kroenke et al., 2019 [49] | 294 veterans with chronic musculoskeletal pain and depression | Randomized trial | Depression and anxiety symptoms | Automated symptom management (ASM), consisting of: web-based self-management modules and interactive voice recorded (IVR) telephone-based clinical assessments | ASM-enhanced collaborative care |
| Meglic et al., 2010 [51] | 45 adults with depression | Randomized trial | Adherence; depression and anxiety symptoms | web-based platform with both patient and provider facing features | Collaborative care alone |
| Ramirez et al., 2016 [43] | 125 adults with type 2 diabetes in a safety-net clinic system with comorbid depression | Post-hoc subgroup analysis | Acceptability | IVR telephone based clinical screenings and follow up assessments | Collaborative care alone and usual primary care |
| Rollman at al., 2018 [46] | 704 adults with depression and/or anxiety | Randomized trial | Quality of life, depression and anxiety symptoms, durability | online CBT course vs online CBT course + Internet support group, both within the context of CCM | Usual primary care |
| Vidyanti et al., 2015 [44] | 284 adults with type 2 diabetes in a safety-net clinic system with comorbid depression | Post-hoc subgroup analysis  Mixed methods | Patient engagement | IVR telephone based clinical screenings and follow up assessments | Collaborative care alone and usual primary care |
| Wu, et al., 2018 [41] | 1406 Adults with type 2 diabetes in a safety-net clinic system with comorbid depression | three-pronged large quasi-experimental comparative effectiveness trial | Clinical outcomes (both depression and diabetes care) and patient satisfaction | IVR telephone based clinical screenings and follow up assessments | Collaborative care alone and usual primary care |
| Zatzick et al., 2015 [50] | 121 adults with PTSD | Randomized trial | PTSD symptoms | IT‐enhanced collaborative care suite including website, mobile app, email and text messaging for health information. | Usual primary care |
